# Effects of Diabetic Ketoacidosis on Visual and Verbal Neurocognitive Function in Young Patients Presenting with New-Onset Type 1 Diabetes

**DOI:** 10.4274/jcrpe.2158

**Published:** 2015-08-31

**Authors:** Ashley B. Jessup, Mary Beth Grimley, Echo Meyer, Gregory P. Passmore, Ayşenil Belger, William H. Hoffman, Ali S. Çalıkoğlu

**Affiliations:** 1 University of North Carolina at Chapel Hill Faculty of Medicine, Division of Pediatric Endocrinology, North Carolina, United States of America; 2 University of North Carolina at Chapel Hill Faculty of Medicine, Department of Psychiatry, North Carolina, United States of America; 3 Georgia Regents University (Formerly Georgia Health Sciences University), Medical Laboratory, Imaging and Radiological Sciences, Georgia, United States of America; 4 Georgia Regents University, Department of Pediatric Endocrinology and Diabetes, Georgia, United States of America

**Keywords:** diabetic ketoacidosis, Cognition, dehydration, neuroinflammation

## Abstract

**Objective:**

To evaluate the effects of diabetic ketoacidosis (DKA) on neurocognitive functions in children and adolescents presenting with new-onset type 1 diabetes.

**Methods:**

Newly diagnosed patients were divided into two groups: those with DKA and those without DKA (non-DKA). Following metabolic stabilization, the patients took a mini-mental status exam prior to undergoing a baseline battery of cognitive tests that evaluated visual and verbal cognitive tasks. Follow-up testing was performed 8-12 weeks after diagnosis. Patients completed an IQ test at follow-up.

**Results:**

There was no statistical difference between the DKA and non-DKA groups neither in alertness at baseline testing nor in an IQ test at follow-up. The DKA group had significantly lower baseline scores than the non-DKA group for the visual cognitive tasks of design recognition, design memory and the composite visual memory index (VMI). At follow-up, Design Recognition remained statistically lower in the DKA group, but the design memory and the VMI tasks returned to statistical parity between the two groups. No significant differences were found in verbal cognitive tasks at baseline or follow-up between the two groups. Direct correlations were present for the admission CO2 and the visual cognitive tasks of VMI, design memory and design recognition. Direct correlations were also present for admission pH and VMI, design memory and picture memory.

**Conclusion:**

Pediatric patients presenting with newly diagnosed type 1 diabetes and severe but uncomplicated DKA showed a definite trend for lower cognitive functioning when compared to the age-matched patients without DKA.

## INTRODUCTION

Type 1 diabetes in children and adolescents is associated with varying degrees of diminished cognitive function, frequently involving memory, but also the subcategories of other neuropsychological domains (1,2). Studies have not always demonstrated differences in academic performance when children with type 1 diabetes are compared to siblings or classmates, suggesting that cognitive deficiencies may be subtle (3). While the pathogenesis of cognitive decline in patients with type 1 diabetes is not well understood, the pathogenesis is likely multifactorial based on numerous potential perturbators that are present during the crisis of diabetic ketoacidosis (DKA). Early age of onset (4), chronic hyperglycemia based on hemoglobin A1c (HbA1c) (5), the presence of microvascular disease (6) and decreased white matter volume have been associated with cognitive deficits (7). Diagnosis before the age seven is associated with structural and functional changes in the brain (7). Studies vary regarding the relationship between significant hypoglycemia in type 1 diabetic children/adolescents and a deleterious effect on cognition (8).

DKA is an acute metabolic and immunologic crisis that occurs in approximately 25 percent of children in the United States with new-onset type 1 diabetes (9). This life-threatening emergency results from a deficiency of insulin and insulin resistance, systemically (10) and in the brain (11). In addition, the endocrine/metabolic decompensation of DKA involves an increase in the counter-regulatory hormones (12), resulting in progressive hyperglycemia, hyperosmolar dehydration (13), ketoacidosis and the potential for serious electrolyte imbalances (14). Increased secretion of cortisol, a counter-regulatory hormone and potential neurotoxin, increases the prevalence of cognitive impairment in depression (15), an under-recognized co-morbidity of type 1 diabetes in children and adolescents (16). Glucocorticoid-mediated, reduced hippocampal neurogenesis and decreased cognitive function have been reported in the streptozotocin (STZ) rat (17). Of importance, the reduced hippocampal neurogenesis in STZ mice can be reversed by antidepressant treatment (18). Also, a positive correlation of C-reactive protein (CRP) with depression, unrelated to type 1 diabetes, in children and adolescents supports an interaction of emotional stress, systemic inflammation and neuroinflammation (19).

In addition to a neurotoxic effect of glucocorticoids, support for the physiologic stress of DKA and its treatment is also associated with a systemic increase of CRP (20) thus supporting roles for both physiologic and psychological stress in the pathogenesis of cognition and depression.

A recent cross-sectional retrospective study using a computer version of published tasks reported that type 1 diabetic children and adolescents who had a history of uncomplicated [no clinical cerebral edema (CE)] DKA scored lower in cognitive memory tasks of both immediate event-spatial position and event-color associations compared to an age-matched group without a history of DKA (21). In another cross-sectional retrospective study, children with type 1 diabetes were tested two to four weeks after either DKA or hypoglycemia, using the Stanford-Binet (fourth edition) and recordings of event-related potential tests and were compared to age-and sex-matched control children without type 1 diabetes. This DKA group also had a cognitive deficit (22).

In this study, we aimed to investigate the contribution of DKA to the neurocognitive changes observed in children with type 1 diabetes. To avoid the uncertainties of retrospective information and its potential influence in the pathogenesis of cognitive deficits caused by: 1) the degree and duration of hyperglycemia; 2) the frequency/degree of hypoglycemia; 3) potential contribution of macro-and microangiopathic changes in long-term diabetes; and 4) the comorbidity of depression, we conducted this study in newly diagnosed children and adolescents presenting with and without DKA and prospectively administered a standard and well-validated neurocognitive test battery at two points in time. This test battery evaluated: visual planning and memory, verbal memory and expressive language. The primary outcome was the difference in cognitive function based on this test battery.

## METHODS

Patients between the ages of 7 and 18 years presenting to the North Carolina Children’s Hospital with new-onset type 1 diabetes were considered for participation. Exclusion criteria included non-English speaking patients or parents (testing materials were printed in English), chronic medication use (except hormone replacement with levothyroxine), developmental delay, learning difficulties or neuropsychological conditions as reported by parents. Fourteen patients were invited to participate between November 2011 and December 2012. DKA was managed by the pediatric intensive care unit per usual protocol, including intravenous insulin and fluid hydration.

The study protocol was approved by institutional review board. Patients assented to the study along with parents’ consent. All procedures performed in studies involving human participants were in accordance with the ethical standards of the institutional and/or national research committee and with the 1964 Helsinki declaration and its later amendments or comparable ethical standards.

The initial cognitive tests were performed prior to hospital discharge. If DKA was present, initial testing occurred at least 24 hours after DKA resolution (defined as pH≥7.32 and CO2≥18 meq/L) and transfer to the pediatric ward. Follow-up testing (T2) was eight to twelve weeks after the diagnosis of type 1 diabetes during a follow-up visit to the Pediatric Diabetes Clinic. This follow-up testing battery consisted of the same cognitive tasks administered at the initial testing as well as a shortened measure of intelligence using the Wechsler Abbreviated Scale of Intelligence (WASI-IQ).

### Measurements and Instruments

Blood chemistries were collected on admission for each patient. Blood glucose was monitored prior to cognitive tests that were administered in the morning when possible. Patients completed a one-hour testing battery administered by a psychologist. The initial testing battery consisted of a Mini-Mental Status Exam (MMSE) to evaluate for alertness prior to other testing. This was followed by standard neurocognitive tests of visual and verbal cognitive tasks. The visual planning and memory tasks used the following instruments: a) Wide range assessment of memory and learning, second edition (WRAML-2) for design memory, design recognition, picture memory and visual memory index (VMI); b) Stanford-Binet for bead memory, c) Wechsler Intelligence Scale for Children-IV (WISC-IV) for forward spatial span and backward spatial span and d) Delis Kaplan Executive Function System (DKEFS) for design fluency. The verbal memory and expressive language tasks used: a) Wide range assessment of memory and learning, second edition for verbal learning, verbal learning recall and verbal learning recognition; b) WISC-IV for digit span; and c) DKEFS for verbal fluency, with subtests of letter fluency and category switching.

### Statistical Analysis

Standard scores and composite scores were determined for the DKA and the non-DKA groups. A Mann-Whitney U test of group medians was used to test for statistical differences between the DKA and non-DKA group’s demographics and admission values for HbA1c, heart rate (HR) for degree of dehydration, pH, bicarbonate (CO2); MMSE at baseline cognitive testing and WASI-IQ at follow-up cognitive testing. Statistical differences between the two groups at baseline and follow-up testing on the cognitive tasks for visual and verbal variables were established using Mann-Whitney U test of group medians. Effect size differences (ES-d) for DKA and non-DKA across the cognitive tasks for the baseline and follow-up testing were also calculated. Chi-square analysis was used to identify association between cognitive task score changes from baseline to follow-up and DKA/non-DKA patient status. Correlations were calculated between cognitive tasks measured at baseline (T1) with chemistries obtained at admission and between cognitive tasks at baseline or follow-up (T2) with pre-test blood glucose.

## RESULTS

All patients had uneventful recoveries. No patient required mannitol for CE or experienced blood glucose (BG) <70 mg/dL during hospitalization. Three of 14 invited patients declined to participate. Three of the four patients with DKA and five of the seven patients without DKA completed follow-up testing. Patients with DKA underwent baseline testing 29-34 hours after DKA resolution. Patients’ admission parameters and demographics are in Table 1. The DKA group presented with median labs at diagnosis consistent with DKA. Median HR in the DKA group was significantly higher than in the non-DKA group: (U=25, p=0.0472). Neither group reached the clinically significant BRIEF-Global Executive Composite (GEC) score of 70, which is suggestive of lower executive functioning. Table 2 shows mean (standard deviation) and median (range) of the standard scores of all cognitive tasks at baseline (T1) and follow-up (T2) testing.

At baseline, the DKA group scored lower on several visual tasks compared to the group without DKA.

At follow-up, design recognition in the DKA group remained statistically lower than the non-DKA group; all of the other visual tasks achieved or maintained statistical parity between the two groups. There were no significant differences between the DKA and non-DKA groups at baseline or follow-up testing for any of the verbal tasks (Table 2). The correlation determinations (Table 3) show statistically significant direct relationships between several admission chemistries and baseline measurements of both visual and verbal cognitive tasks. Correlations at baseline testing of both visual and verbal cognitive tasks with pre-test BGs indicate inverse correlations. At follow-up, the visual cognitive task of picture memory maintains its inverse correlation with pre-test BG.

Last, chi-square analysis for association of the number of cognitive score that increases between baseline and follow-up showed a significant association (χ2=3.472, p=0.0312) for the DKA group. Similarly, the cognitive variables, VMI and digit span, had significant inverse correlations with the DKA group (r=-0.6635, p=0.0260 and r=-0.7221, p=0.0121, respectively), meaning that lower cognitive scores correlate with the DKA group.

## DISCUSSION

This is the first prospective study to report acute cognitive deficits in newly diagnosed children and adolescents with type 1 diabetes following correction of uncomplicated (no clinical CE) DKA. Our data support a neuronal insult during the metabolic and immunologic crisis of DKA and/or its treatment that results in acute and possibly long-term cognitive deficits. We recorded statistically lower median baseline (T1) task scores in three of the eight visual cognitive tasks tested in the newly diagnosed DKA group in comparison to an age-matched newly diagnosed non-DKA group. Whether the improvement in design memory and VMI scores three months (T2) after the correction of DKA are final new baselines is unknown, since the follow-up tasks were performed at a time when brain remodeling was possibly occurring (23). These longitudinal results differ from the retrospective, cross-sectional, solitary time point studies that reported color and position associations in children and adolescents with a history of DKA that were believed to indicate permanent cognitive deficits (21). In contrast, the follow-up (T2) testing of the median task score for design recognition in the DKA group decreased further from the baseline; however, for the non-DKA group, the score remained constant. This is in keeping with previous speculation that the neurotoxic milieu of DKA initiates a greater insult than cognitive deficit with only hyperglycemia. A pattern of decreasing cognition with time is also in keeping with the proton-magnetic resonance spectroscopy (MRS) study of recurrent episodes of DKA in a teenage patient, where the NAA/Cr ratio (neuronal insult) decreased during each DKA treatment, followed by a lesser recovery after correction of the second episode of DKA; cognitive testing was not reported (24).

The combined admission CO2 and pH of the DKA and non-DKA groups versus the T1 results of the visual and verbal cognitive tasks (Table 3) showed significant direct correlation with six baseline tasks; design recognition, design memory, picture memory and VMI as did verbal learning and category fluency. These correlations are in keeping with the association of an acute neuronal insult in close proximity to the T1 cognitive tasks and also supportive of the direct correlation between the admission level of consciousness and admission pH at the time of DKA (25). However, it should be noted that we found no correlation between the Glasgow coma score and cognitive testing, nor a difference between the mini-alertness tests prior to cognitive testing of the DKA and non-DKA groups. In addition, the visual tasks of bead memory, picture memory and VMI and the verbal task of digit span had an inverse correlation with the pre-test (T1) BG. An inverse correlation was also present for picture memory and the pre-test (T2) BG. This inverse pre-test BG correlation with cognitive tasks in our study was not present in the study by Ghetti et al (21). While it is not statistically significant, it is important to note that there was a large difference (effect size=2.5) between the admission HbA1c in our DKA and non-DKA groups suggesting that the cases with DKA had longer and/or more severe hyperglycemia at the time of testing. However, no correlation was found between HbA1c levels and cognitive testing in our study. In addition, there was no difference in the HbA1c pre-test values of DKA and non-DKA groups in the study by Ghetti et al (21).

Although the pathogenesis of the cognitive deficiencies associated with DKA requires further study, it has been reported that ketone bodies, by-products of metabolic dysregulation, are capable in-vitro of differentially perturbating cerebral capillary endothelial cells (CCEC) (26) and the increased release of vasoactive peptides such as endothelin-1 (ET-1) (27) and vascular endothelial growth factor (VEGF) (28) that negatively impacts cognition. Hyperlipoproteinemia (29) and toxic tryptophan catabolites (30) are additional by-products of DKA metabolic dysregulation and its treatment and thus candidates to affect cognition.

Also, 3-deoxyglucosone (31) and methylgloxal (32), the highly reactive carbonyl compounds and neurotoxins, are precursors of advanced glycation end-products (AGE) that increase during treatment and up-regulate the inflammatory milieu of DKA (33).

These by-products are unlikely to be the sole candidates of neuronal insult/deficit since the milieu in children and adolescents with severe DKA also increases systemic and cerebral oxidative stress (34,35), SIR (36) and a likely neuronal insult. Systemic inflammatory cytokines, activation of the complement cascade including C5b-9, the membrane attack complex (37) and CRP (20) are part of the innate immune response activated by DKA that increases during treatment. Evidence for an acute neuronal insult by oxidative stress and inflammatory peptides is the immune cytochemistry (ICC) study of autopsied brains of adolescents with type 1 diabetes, where death occurred due to DKA/CE. These cases had histories of chronic poor diabetic control and poor school performance along with significant cerebral expression of oxidative/nitrosative stress and lipid peroxidation (35), as well as an acute inflammatory insult, based on glial and CCEC activation, expression of inflammatory peptides and neuronal deficits (11,38,39). It is important to recognize that the neurotoxic immune phenotype in the brains with fatal DKA/CE is similar not only to the SIR of clinical DKA (36,37), but it also develops over time in type 1 diabetic rodent models, without the acute metabolic and immunologic insults of DKA and the occurrence of CE (40). The latter is in keeping with Hood et al’s clinical study (41) and thus DKA and CE do not appear to be prerequisites for neuronal and cognitive deficits, but rather additional severe insults. A sustained cognitive injury is also suggested based on: 1) the infrequent reports of immediate cognitive deficits following severe DKA (23); 2) the extended and increased systemic concentration of inflammatory cytokines even after correction of DKA (36); 3) the decreased density of neuroprotective GH and IGF-1 neuronal receptors in various brain regions including the hippocampus in DKA/CE (11,38,39), a region that is important for the formation and retrieval of numerous memory systems. The neuroinflammatory phenotype in the BB/W type 1 diabetic rat (40); and perhaps most importantly; 4) the activation of the AGE/receptor for advanced glycation end products (RAGE), an amplifier and perpetuator of oxidative stress and dysregulation of inflammatory pathways in the brain with or without DKA (39,40). A progressive and sustained cognitive injury also supports; 5) The observation that children whose DKA was in the more distant past had a poorer memory performance (21).

Limitations of this study are the small sample size, the short duration of follow-up and the absence of testing non-diabetic children and adolescents. Strengths of the study are that: 1) It is a prospective study involving patients of comparable ages with newly diagnosed type 1 diabetes with and without DKA; 2) It eliminates the confounding factors of hypoglycemia and microvascular disease; 3) It uses standardized cognitive testing; 4) It reports short-term follow-up testing; 5) It extends our insight into the subtleties of the early cognitive deficits in young patients with new-onset type 1 diabetes and DKA; and 6) It shows the importance of longitudinal testing.

Understanding the physiological and functional changes that accompany DKA may contribute to early detections of brain changes associated with DKA, prior to the onset of chronic debilitating effects of type 1 diabetes and may inform about the mechanisms associated with subsequent cognitive decline. Modalities to study the effect of diabetes on the brain have evolved over the years and provide new opportunities not only to examine the impact of DKA on neurocognitive dysfunction but also to understand the underlying mechanisms. Therefore, further studies in larger populations using new static and functional neuroimaging tools are needed. Nevertheless, this study serves to encourage the careful follow-up, preferably in a coordinated care setting, of all children/adolescents with type 1 diabetes, especially those who have had DKA. Whether routine neurocognitive testing can be recommended in children with type 1 diabetes as part of their follow-up is an important question that remains to be answered.

## Figures and Tables

**Table 1:**
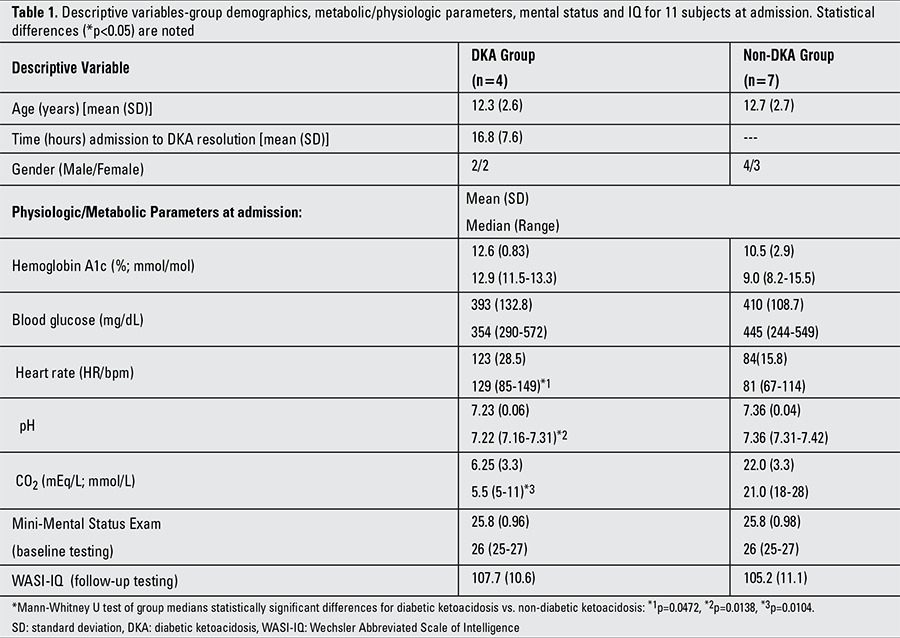
Descriptive variables-group demographics, metabolic/physiologic parameters, mental status and IQ for 11 subjects at admission. Statistical differences (*p<0.05) are noted

**Table 2:**
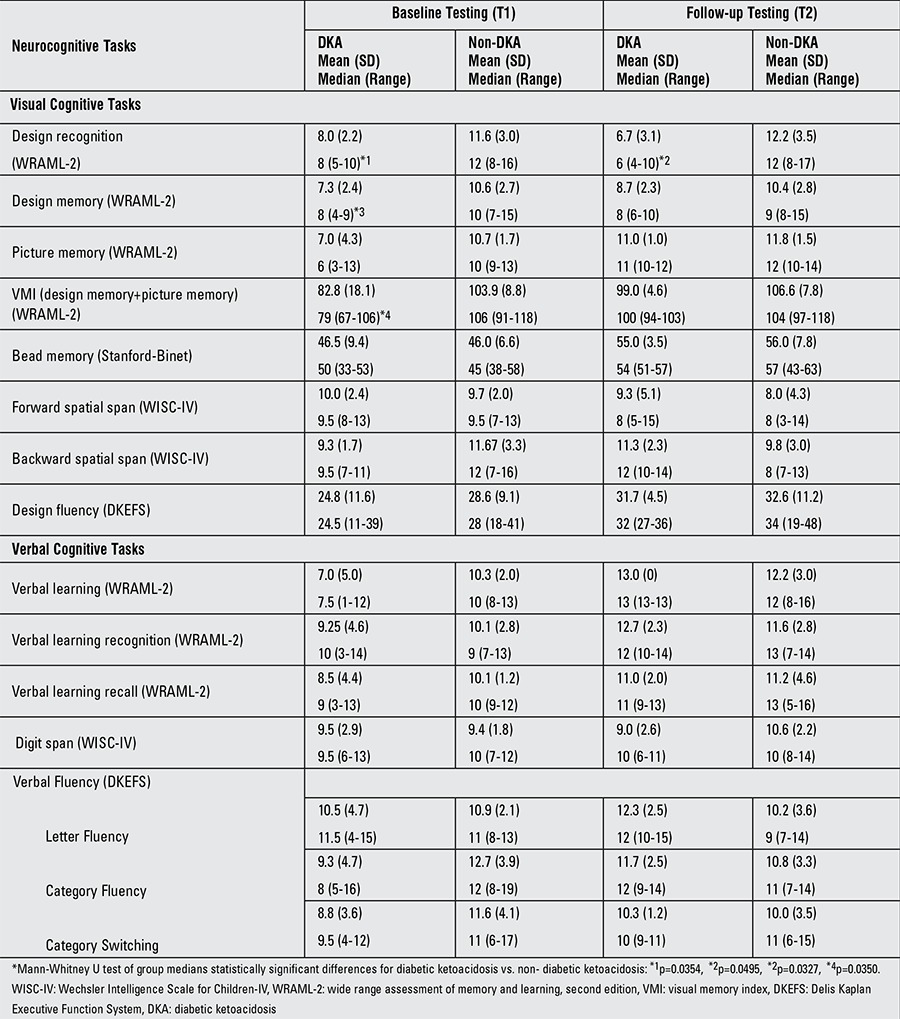
Neurocognitive tasks. Mean (standard deviation) and median (range) of standard scores of all neurocognitive tasks at baseline and follow-up testing for diabetic ketoacidosis and non-diabetic ketoacidosis subjects. Statistical differences (*p<0.05) are noted

**Table 3:**
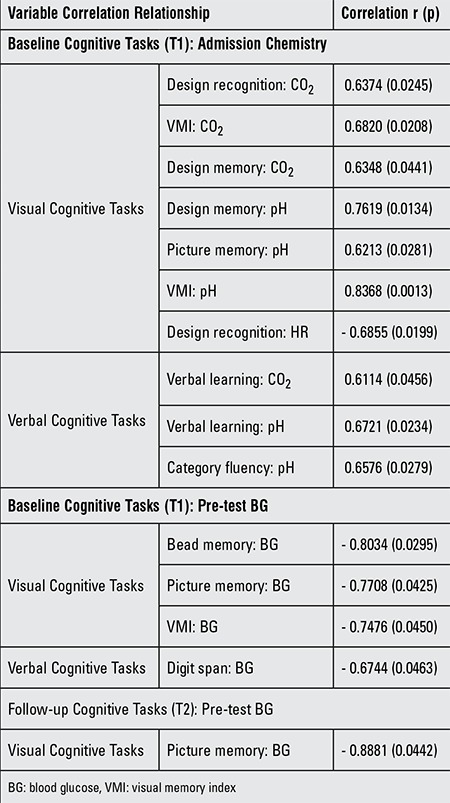
Statistically significant (p<0.05) correlations for neurocognitive tasks measured at baseline (T1) and follow-up (T2), with chemistries and heart rate measured at admission and blood glucose at T1 or T2
